# Rhombencephalitis associated with isolated Zic4-antibodies in Paraneoplastic cerebellar degeneration: a case report

**DOI:** 10.1186/s12883-020-01788-z

**Published:** 2020-05-25

**Authors:** Philipp A. Loehrer, Lars Timmermann, Anika Pehl, Corinna I. Bien, Andreas Pfestroff, David J. Pedrosa

**Affiliations:** 1 Department of Neurology, University Hospital of Gießen and Marburg, Marburg, Germany; 2 Institute of Pathology, University Hospital of Gießen and Marburg, Marburg, Germany; 3 Laboratory Krone, Bad Salzuflen, Germany; 4 Department of Nuclear Medicine, University Hospital of Gießen and Marburg, Marburg, Germany

**Keywords:** Paraneoplastic Cerebellar Degeneration, Zic4, Paraneoplastic Syndromes, Autoimmune Encephalitis, Case Report

## Abstract

**Background:**

Cerebellar degeneration as a consequence of a malignancy is a rare condition most commonly related to the presence of anti-Yo, anti-Hu, and anti-Tr/DNER antibodies. In recent years, several reports have indicated Zinc-finger protein 4 (Zic4) antibodies being associated with paraneoplastic cerebellar degeneration (PCD) in patients with small cell lung carcinoma. However, the prevalence and the significance of Zic4-antibodies may be underestimated due to their co-occurrence with more frequent antibodies such as anti-Hu. A literature review of isolated Zic4 mediated paraneoplastic syndromes yielded 14 cases reporting mainly benign clinical courses when treated early.

**Case presentation:**

We present the case of a 67-year-old woman with progressive Zic4 antibody mediated PCD and rhombencephalitis. Immunomodulatory treatment, including intravenous methylprednisolone, plasmaphereses, and intravenous immunoglobulin (IVIG) was administered. Small cell lung cancer (SCLC) was detected, lobectomy performed and cyclophosphamide started. Despite this considerable therapeutic effort, rhombencephalitis led to defiant dysautonomia.

**Conclusion:**

Paraneoplastic syndromes related to isolated Zic4 antibodies are rare and typically show a benign clinical course. Here, we present the first case of a rapidly progressive isolated Zic4 associated PCD and rhombencephalitis. Despite considerable therapeutic efforts, the patient passed away on autonomic dysfunction, highlighting the significance of Zic4 associated disease.

## Background

Paraneoplastic cerebellar degeneration (PCD) is one of the most frequent paraneoplastic presentations [1]. It is characterized by a subacute onset of symmetrical limb and truncal ataxia, dysarthria, and nystagmus. The symptoms can be preceded by a prodromal phase including fever, headache, nausea, and vomiting, whereby progression to pancerebellar dysfunction occurs within weeks [1]. Symptoms of PCD can antecede tumor diagnosis in more than 50% of the patients depending on the tumor and associated antibody [2, 3]. Initial brain imaging is usually normal, whereas cerebrospinal fluid (CSF) shows pleocytosis, elevated protein levels, and intrathecal IgG synthesis [4]. Malignancies commonly associated with PCD are small cell lung cancer (SCLC, 33%), ovarian carcinoma (25%), and Hodgkin lymphoma (15%) [4].

The pathogenesis of PCD is attributed to an autoimmune response elicited by the underlying malignancy when proteins restricted to immune privileged neurons are presented by the tumor [4]. In 60% of the cases, onconeuronal antibodies (targeting intracellular proteins) are identified, although associations with antibodies to cell surface proteins like voltage-gated calcium channels (VGCC) have been described [4]. Identifying the respective antibody is important, since presence of a well-characterized antibody helps to establish the diagnosis of PCD and specific associations between antibody and cancer type exist. Among the most prevalent antibodies in PCD are anti-Yo, anti-Hu, and anti-Tr/DNER antibodies. Anti-Yo antibodies (also known as anti-Purkinje cell antibody 1) are directed against Purkinje-cells and typically associate with breast or ovarian cancer as well as a primarily isolated cerebellar syndrome. Anti-Hu antibodies (also known as antineuronal nuclear antibody-type 1, ANNA-1) are attributed to SCLC and the clinical presentation of an encephalomyelitis. Anti-Tr/DNER (delta/notch-like epidermal growth factor-related receptor) antibodies associate with Hodgkin lymphoma and conduct to an isolated cerebellar syndrome.

In the last decades, multiple antibodies have been linked with PCD including Zinc-finger protein 4 (Zic4) antibodies [4, 5]. Zic4 antibodies are considered onconeuronal antibodies with the zinc finger domain of the intracellular transcription factor Zic4 as the target antigen [6]. Due to the intracellular location of Zic4, antibody associated autoimmunity is potentially T-cell mediated, although exact mechanisms are unknown [5]. Zic4 antibodies hence serve as a biomarker for the syndrome and underlying tumor (see below). The zinc-finger protein of the cerebellum (ZIC) family comprise five transcription factors involved in cerebellar development and maturation [7]. Each factor consists of five Cys2His2 zinc-finger domains and is encoded by one of five *ZIC*-genes, all of which remained highly conserved throughout evolution [7]. Aberrant *ZIC*-gene expression during embryogenesis may entail Dandy-Walker malformation (Zic1 and Zic4), neural tube defects (Zic2), holoprosencephaly (Zic2), and heterotaxy syndrome (Zic5) [7]. In adults, contrarily, *ZIC*-protein family alterations primarily manifest as cerebellar dysfunction.

Patients harboring Zic4 antibodies commonly develop PCD and 92% have SCLC [6]. However, most patients with Zic4 antibodies have concomitant anti-Hu or CRMP5 (collapsin response mediator protein 5) antibodies and present with various additional paraneoplastic syndromes, obscuring the clinical significance of Zic4 antibodies [6, 8]. Isolated Zic4 antibodies, contrarily, appear to be associated with benign paraneoplastic syndromes when treated early. Here, we present the first case of isolated Zic4 antibodies associated with PCD and rhombencephalitis, which subsequently led to fatal dysautonomia, adding important information on how this particular antibody can affect the central nervous system.

## Case presentation

A 67-year-old woman with no relevant past medical or family history was admitted to our emergency department due to progressive staggering vertigo. Ten days prior to admission, the patient had noted unsteadiness of gait and inadequate coordination of her extremities along with truncal instability. In the neurological examination on admission, the patient presented with bilateral dysmetria but especially severe truncal ataxia disabling her to walk. Examination of vertical eye movements revealed upbeat nystagmus, whereas pupillary function, horizontal smooth pursuit, and the vestibulo-ocular reflex were unremarkable. Further neurologic examination was normal, as were the initial laboratory results.

**Fig. 1:**
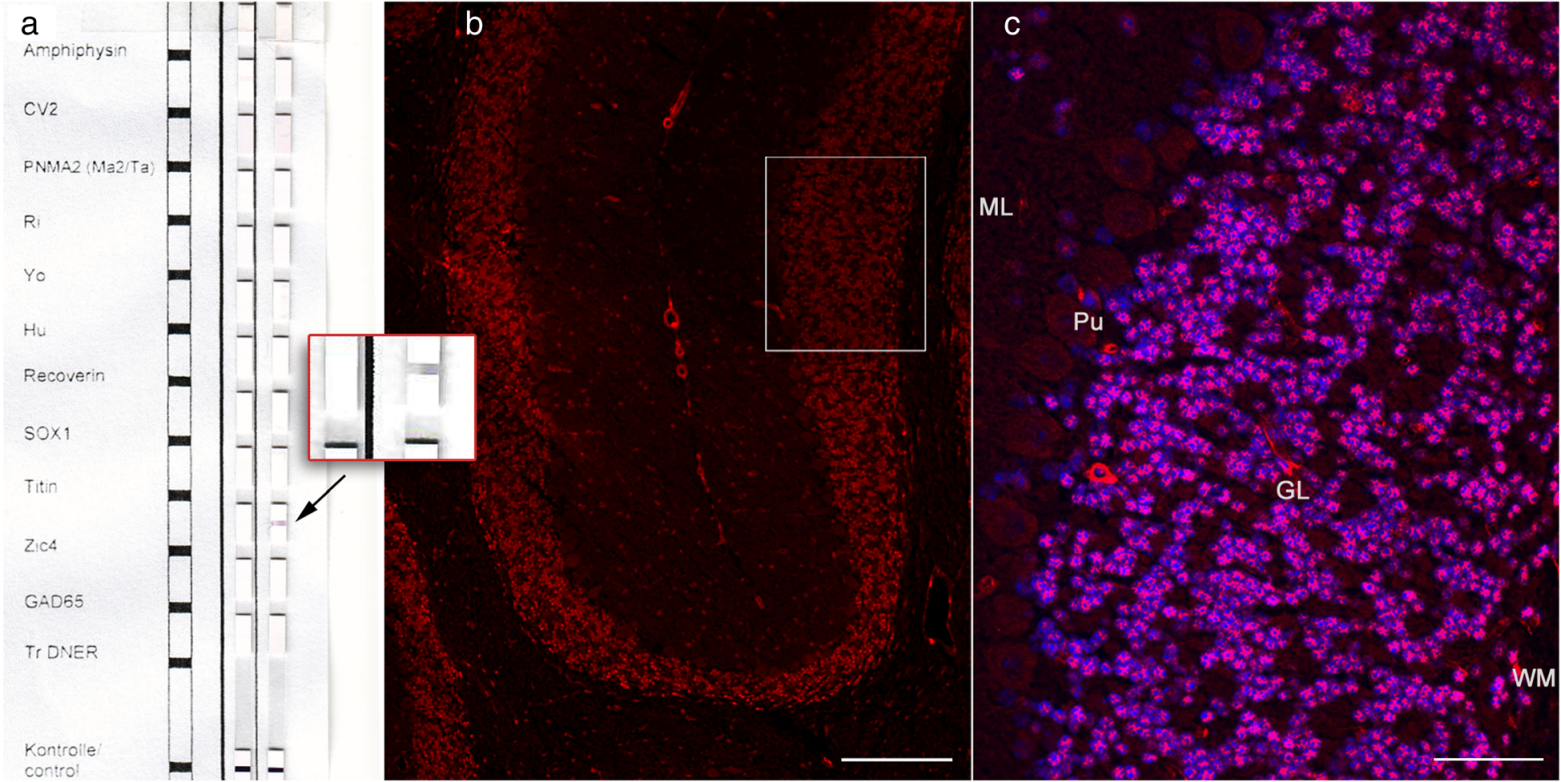
Immunoblot and Immunofluorescence Test on Mouse Cerebellum. Demonstration of Zic4 antibodies in serum (the same results were obtained with cerebrospinal fluid, not shown). **a** On an immunoblot, 1:100 diluted patient serum produces on the right lane a band with Zic4 only (arrow and insert), not with any other antigen (Euroline DL 1111–1601-4 G, Euroimmun, Lübeck, Germany). The left lane was incubated with a control serum. **b** Incubation of patient serum, diluted 1:20, with unfixed mouse cerebellum (Euroimmun, Lübeck, Germany). Patient’s immunoglobulin G (IgG) binds in a Zic-4-typical pattern to the cerebellar granular cells (cf. Fig. 1 in Bataller et al., 2002 [5]). Bound antibodies are visualized by an anti-human-IgG antibody coupled to a red fluorochrome. Original magnification × 100. Bar: 100 μm. The area in the rectangle is shown in (C). **c** Original magnification × 400. Bar: 25 μm. Zic-4 IgG-antibodies bind to the nuclei of the granular layer and spare the nucleoli. Nuclear counterstaining with Hoechst 33342 1:10.000 in blue. ML = molecular layer; Pu = Purkinje cell layer; GL = granular layer; WM = white matter

**Fig. 2:**
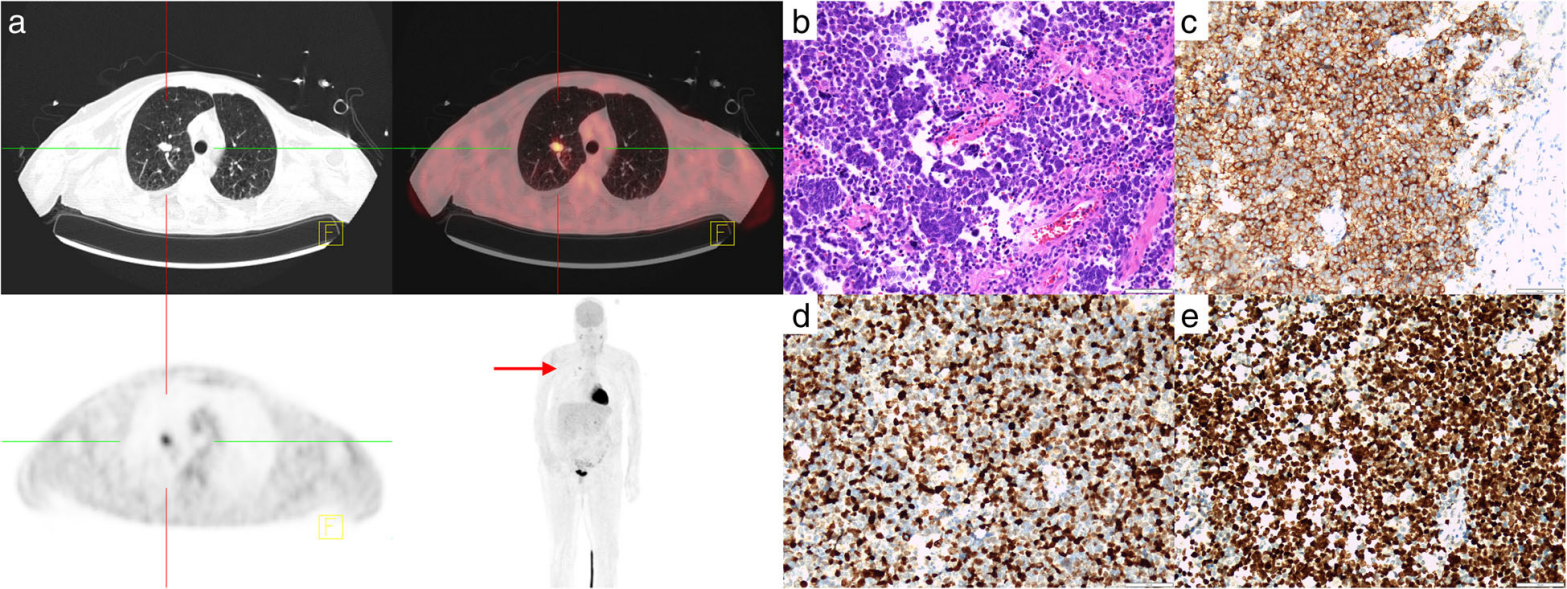
18F-FDG-PET Findings and SCLC Histology. **a** FDG-avid lesion in the right upper pulmonary lobe (red arrow) and non-specific uptake in cervical lymph nodes as a consequence of a mild inflammation. **b** Hematoxylin and eosin (HE) staining (magnification × 200), shows the tumor with typical basophil small cells with sparse cytoplasm and irregular nuclei. **c** Immunohistochemical (IH) staining for Synaptophysin (magnification × 200). Tumor tissue stains positive (brown), confirming neuroendocrine differentiation. **d** IH staining for Ki67 (magnification × 200) marks proliferating cells in a tumor. Proliferation rate was up to 70% in this case. **e** IH staining for TTF-1 (magnification × 200), shows the typical nuclear staining of cells of pulmonary origin

Magnetic resonance imaging (MRI) of the head, including contrast enhanced scans, revealed no pathological findings beyond minor nonspecific vascular white matter hyperintensities on fluid attenuated inversion recovery (FLAIR) sequences. Especially no abnormalities in the brainstem or the cerebellum were present. Cerebrospinal fluid (CSF) showed a pleocytosis (97/mm^3^) with positive oligoclonal bands. Consecutive serum and CSF analyses were negative for active infectious or systemic autoimmune causes. An immunoblot for intraneuronal antibodies revealed Zic4 antibodies in serum and CSF (coverage: Table 1 and Fig. 1). A combined positron-emission tomography and computed tomography (PET-CT) revealed substantial 18F-fluorodeoxyglucose (FDG) uptake in the right upper pulmonary lobe consistent with lung cancer (Fig. 2**.A**). Subsequent immunohistochemistry after lobectomy confirmed small cell lung carcinoma (SCLC, Fig. 2**.B-E**), insofar as that TTF-1 (thyroid transcription factor-1) evidenced a pulmonary origin (Fig. 2**.E**) and Synaptophysin-staining demonstrated neuroendocrine differentiation (Fig. 2**.C**). Moreover, a fraction of about 70% of Ki67-positive cells were indicative of high proliferation rates in this tumor (Fig. 2**.D**). The patient had previously admitted history of long-term cigarette smoking.

**Table 1:** Antibodies assessed in this Case

Immunoblot	Cell-based assays	Radioimmunoassay
Amphiphysin	GAD65	VGCC
CV2/CRMP5	NMDAR	
Ma2/Ta	GABABR	
Ri	IgLON5	
Yo	AMPAR2	
Hu	DPPX	
Recoverin	LGI1	
Sox1	CASPR2	
Titin	Glycinereceptor	
Zic4	mGluR5	
DNER/Tr	mGluR1

Under the diagnosis of a paraneoplastic cerebellar degeneration (PCD), intravenous methylprednisolone 1000 mg q.d. was administered over five days, followed by five sessions of plasma exchange. In consequence of increasing respiratory distress and incapacitating opsoclonus-myoclonus syndrome, we had come to an agreement with the patient before to initiate sedation and mechanical ventilation not least because of the pending lobectomy.

After tumor resection, clinical presentation remained unchanged, so that intravenous immunoglobulin (IVIG) were administered for five additional days and cyclophosphamide therapy was started. The patient was discharged to rehabilitation in a cardiopulmonary stable but reduced overall state requiring mechanical ventilation. Regrettably, she deceased six weeks later due to cardiac arrest.

## Discussion and conclusions

This is the first report of isolated Zic4 antibodies associated with rhombencephalitis leading to fatal dysautonomia. In reviewing the literature, a Medline search returned only 14 cases with isolated Zic4 antibodies (Table 2). In the majority of these reports early immunomodulatory treatment conduced to a benign clinical course. Only a single case reported a similarly rapid and clinically severe affection based on coincident spontaneous Creutzfeldt-Jacob disease (CJD) [9]. The authors thereby speculated about autonomic dysfunction and respiratory failure in CJD due to prion deposition in respiratory nuclei. In our patient, however, CJD was ruled out and early immunomodulatory treatment resulted in limited clinical improvement over the entire course. Particularly autonomic dysfunction with cardiac instability and respiratory insufficiency were defiant. In contempt of numerous interdisciplinary tertiary hospital diagnostics and considerable therapeutic efforts, no other pathology was traceable, indicating exclusive paraneoplastic cause. Nevertheless, we acknowledge that presence of other, yet unidentified antibodies cannot be ruled out. In this context, we refer to a recent report describing autoantibodies against Kelch-like-protein-11 (KLHL11-ab) causing a paraneoplastic brainstem and cerebellar syndrome resembling the clinical appearance of our patient [13]. Despite an unavailable specific test to-date, tissue-based assays were not indicative of the presence of KLHL11-ab in our case.

**Table 2:** Summary of Case Reports of isolated Zic4-Antibodies

Author	Age/Sex	Associated Tumor	Primary syndrome at Diagnosis	Symptoms	Treatment	Treatment response
Current Study	67/F	SCLC	Brainstem, PCD	Opsoclonus-Myoclonus Syndrome, Dysautonomia	Methylprednisolone, Plasmapheresis, IVIG, Lobectomy, Cyclophosphamide	none
Salazar et al. 2018 [9]	70/M	None, sCJD	Brainstem, PCD	Dementia, Ataxia, Myoclonus, Dysautonomia	Methylprednisolone	none
Eye et al. 2018 [10]	94/F	DLBCL, SMM	PCD	Downbeat Nystagmus, alternating Skew Deviation, Gait Ataxia	Chemotherapy, Rituximab	Improvement but persisting symptoms
Aydin et al. 2018 [11]	40/F	Ductal breast cancer	SSN	Pain, Asymmetric Numbness	n/a	Improvement but persisting symptoms
Kerasnoudis et al. 2011 [12]	60/F	Ovarian adenocarcinoma	PCD	Progressive Gait Ataxia, Dysarthria	Methylprednisolone, Ovariectomy, Chemotherapy	Complete clinical remission
Sabater et al. 2008 [8]	n/a	SCLC	PCD	n/a	n/a	n/a
Bataller et al. 2004 [6]	67^a^/ 8: M, 1: F	8: SCLC 1: no tumor	7: PCD 1: PCD + LE 1: LEMS	Cerebellar Ataxia Cerebellar Ataxia, Cognitive Dysfunction Myasthenia	n/a	n/a

^a^median age

Irrespective of the divergent clinical course, our case is conforming to previous reports suggesting a link between PCD and Zic4 antibodies. Their presence has most frequently been attributed to SCLC as in our patient [6, 8]. Nevertheless, a source of uncertainty as for the clinical manifestation related to these antibodies may be the co-occurrence with other antibodies like Anti-Hu- or CRMP5-antibodies [6]. Hence, in the first comprehensive case series, Bataller et al. indicated that in 215 patients with paraneoplastic neurologic disorders, 49 showed Zic4 antibodies but only nine patients its exclusive manifestation [6].

In conclusion, we present a rapidly progressive Zic4 antibody related PCD resulting from local SCLC. Despite considerable therapeutic efforts, the patient passed away within few weeks after symptom-onset on autonomic dysfunction. This underlines the significance of isolated Zic4 antibody associated paraneoplastic syndrome and illustrates how this particular autoimmune disorder can affect the central nervous system.

## Data Availability

Data sharing is not applicable to this article as no datasets were generated or analyzed during the current study.

## Abbreviations

AMPAR2: α-amino-3-hydroxy-5-methyl-4-isoxazolpropionic acid receptor
ANNA-1: antineuronal nuclear antibody-type 1
CASPR2: Contactin-associated protein 2
CRMP5: collapsing response mediator protein 5
CSF: cerebrospinal fluid
CV2/CRMP5: collapsin response mediator protein 5
DLBCL: diffuse large B-cell lymphoma
DNER: Delta/notch-like epidermal growth factor-related receptor
DPPX: Dipeptidyl-Peptidase-like Protein-6
FDG: fluorodeoxyglucose
FLAIR: fluid attenuated inversion recovery
GABABR: γ-aminobutyric acid-B receptor
GAD65: glutamic acid decarboxylase 65 kDa
GL: granular layer
HE: hematoxylin and eosin
IgG: Immunoglobulin protein, subclass G
IgLON5: immunoglobulin-like cell adhesion molecule 5
IH: immunohistochemical
IVIG: intravenous immunoglobulin
LE: limbic encephalitis
LEMS: Lambert-Eaton myasthenic syndrome
LGI1: Leucine-rich, glioma inactivated 1
mGluR1/5: metabotropic glutamate receptor 1/5
ML: molecular layer
mm^3^: cubic millimeter
NMDAR: N-methyl-D-aspartate receptor
PCD: paraneoplastic cerebellar degeneration
PET-CT: combined positron-emission tomography and computed tomography
Pu: Purkinje cell layer
q.d.: quaque die
Ri: Nova 1 or ANNA-2 (anti-neuron-specific cell nuclear antibodies)
(s)CJD: (spontaneous) Creutzfeldt-Jacob disease
SCLC: small cell lung cancer
SMM: smoldering multiple myeloma
Sox1: anti-glia nuclear antibody
SSN: subacute sensory neuropathy
TTF-1: thyroid transcription factor-1
VGCC: voltage-gated calcium channel
Yo: anti-PCA-1 (Purkinje cell antigen 1)
ZIC: zinc-finger protein of the cerebellum
